# Multilevel obstacles to heart-healthy living in the context of Ischemic heart disease: A qualitative study

**DOI:** 10.1371/journal.pone.0338298

**Published:** 2025-12-09

**Authors:** Bardia Amidi, Narges Naderi, Javad Yoosefi Lebni, Arash Amin

**Affiliations:** 1 Student Research Committee, Lorestan University of Medical Sciences, Khorramabad, Iran; 2 Health Policy Research Center, Institute of Health, Shiraz University of Medical Sciences, Shiraz, Iran; 3 Social Determinants of Health Research Center, Lorestan University of Medical Sciences, Khorramabad, Iran; 4 Department of Cardiology, School of Medicine, Shahid Madani Hospital, Lorestan University of Medical Sciences, Khorramabad, Iran; Dow University of Health Sciences, PAKISTAN

## Abstract

**Background:**

Ischemic heart disease (IHD) is a major contributor to global morbidity and mortality. Lifestyle modifications play a central role in preventing and management of IHD, yet patients face barriers to engaging in healthy behaviors. This study explored these barriers among IHD patients in Lorestan Province, Iran.

**Methods:**

A qualitative study using conventional content analysis was conducted. Semi-structured interviews were held with 31 IHD patients and 8 key informants (3 cardiologists, 2 cardiology residents, and 3 ward nurses), selected through purposive sampling. Data were analyzed using MAXQDA-2020 based on the Granheim and Lundman approach. Guba and Lincoln’s criteria were applied to ensure trustworthiness.

**Results:**

Data analysis led to the identification of four main categories, 17 subcategories, and 413 primary codes. The categories identified included socio-cultural barriers, such as taboos surrounding women’s sports, misconceptions about sports, fatalism, unhealthy dietary patterns and beliefs, and patterns and beliefs regarding the use of addictive substances. Economic barriers included the economic crisis and the high cost of a healthy lifestyle. Individual barriers included a lack of prioritization of personal health, unhealthy food preferences, lack of access to facilities and conditions for a healthy lifestyle, personality traits, and low health literacy. Healthcare-medical barriers included communication challenges in healthcare, management and infrastructure challenges in healthcare services, gaps in patient education and healthcare delivery, and the perceived insignificance of lifestyle recommendations by patients. These interrelated barriers highlight the compounded difficulties IHD patients face in adopting and sustaining a healthy lifestyle.

**Conclusion:**

This study identified multifaceted lifestyle modification barriers in IHD patients including sociocultural, economic, individual, and healthcare system factors. These necessitate community-based intervention, financial support for healthy living, tailored education, and health system reform to include systematic lifestyle counseling in regular care. Future studies are needed to evaluate the feasibility of these interventions to improve long-term health outcomes in IHD patients.

## Introduction

Ischemic heart disease (IHD) is the most common form of cardiovascular disease [1]. IHD is one of the critical public health concerns of our time, becoming a leading cause of morbidity and mortality in nations worldwide [2]. Iran’s national age-standardized IHD disability-adjusted life years (DALYs) decreased by 33.7% from 1990 to 2019, whereas death rates declined by 22% over the same period [3]. However, IHD is still the primary cause of DALYs and death in Iran [4].

Unhealthy diet, inactivity, smoking, and obesity are modifiable risk factors that play important roles in the incidence and progression of IHD [5,6]. Research has shown that modification of lifestyle through the consumption of a heart-healthy diet, regular physical activity, maintaining a healthy weight, avoiding tobacco and opium, managing mental stress, and reducing alcohol consumption can greatly reduce the occurrence of complications and mortality due to IHD [6–9]. Optimal control of these factors has been associated with improved cardiovascular outcomes, reduced hospitalization rates, and improved quality of life among IHD patients [10–12]. However, barriers to lifestyle change, such as physical, psychological, social, practical, habitual, and motivational barriers, have been demonstrated to limit success in eliminating related risk factors and maintaining a healthy lifestyle in patients with IHD [13–17].

Despite the enormous burden of IHD on Iranian society and the crucial role of modifiable risk factors and lifestyle modifications in its prevention and development, IHD patients face many challenges in implementing and sustaining healthier lifestyle practices. Understanding these challenges is necessary for designing effective intervention programs. Qualitative research is a valuable method for exploring patients’ experiences, perceptions, and challenges [18], and can be helpful in achieving this understanding. Thus, we conducted this study with the aim to explore the barriers that IHD patients face in adopting healthier lifestyles through a qualitative approach. We also conducted interviews with key informants to identify the observed barriers in IHD patients.

## Methods

### Design

The present study was conducted on IHD patients and key informants in Lorestan province in western Iran in 2025, using a qualitative approach and conventional content analysis. Lorestan Province was chosen as the study setting because of its unique socio-cultural and environmental characteristics that can potentially affect lifestyle behaviors. Understanding these local dynamics is essential for designing context-specific interventions aimed at improving cardiovascular health in the region.

### Participants

The participants of the study consisted of two groups. The first study group included IHD patients, and the second group consisted of key informants, including cardiologists, cardiology residents, and cardiology ward nurses. The inclusion criteria for patients with IHD included not following a healthy lifestyle regarding IHD, living in Lorestan province, the ability to communicate verbally, and willingness to participate in the study. The inclusion criteria for the key informants included having working experience or specific knowledge about IHD patients and the willingness to participate in the study. The exclusion criteria for both groups were failure to complete the interview and unwillingness to record the interview process.

### Sampling and recruitment

Participant recruitment for this study began on January 16, 2025, and concluded on March 3, 2025. In this study, the participants were first identified and chosen through a purposive sampling method. A list of IHD patients was initially compiled by visiting Shahid Madani Hospital. The patients were subsequently contacted via phone to assess whether they had a healthy lifestyle. A healthy lifestyle was defined by the consumption of a well-balanced diet rich in fruits, vegetables, and whole grains, and a restriction on saturated fats and sugars; regular physical exercise, with a minimum of 150 minutes of moderate-intensity physical exercise per week; abstaining from tobacco use; abstaining from opioid use; limiting or abstaining from alcohol consumption; and effective stress management with the use of proper coping mechanisms. For those who did not have a healthy lifestyle, oral consent was obtained for participation in the study, and the time and place for in-person interviews were coordinated.

### Data collection

Face-to-face, semi-structured interviews were conducted with the participants to collect the data (39 interviews). Initially in each interview, the interviewer explained their study field, job, and role in the research to create effective communication and build trust. The interviewer subsequently explained the study’s objectives and procedure and how the results would be reported. If the participants provided their written consent, the interview began in the presence of only the researcher and the interviewee. The interview questions were developed collectively by the research team through multiple rounds of discussion and refinement, with the support of an extensive review of the literature. Five pilot interviews were conducted to verify that the questions could capture participants’ views and experiences. The analysis of the pilot interviews was used to further refine the interview guide, which is available as Supplementary File 1. First, participants were asked demographic questions, which were followed by core questions provided in the interview guide. Additionally, based on the participants’ answers, follow-up and probing questions were posed to delve deeper into their perspectives. The key informants were interviewed after the patients were interviewed. The interviews were audio-recorded, and field notes were taken during the sessions to capture non verbal cues such as tone of voice, pronunciation, laughter, crying, and speech pauses. All of the interviews were conducted in locations chosen by the participants, thus ensuring a peaceful and private atmosphere. The interviews lasted an average of 36 minutes, with the longest interview lasting 54 minutes and the shortest one lasting 25 minutes. From the very first interview, coding and data analysis were started, and codes were frequently compared and examined. During the 27th interview with IHD patients and the 6th interview with key informants, the codes became repetitive and no new codes appeared, indicating theoretical saturation. To be assured that saturation was reached, the researchers conducted four more interviews with IHD patients and two more with key informants. Then, the researchers came to the conclusion that ultimate saturation had been reached and that more interviews would not add any new findings to the research.

### Data analysis

The process of categorizing and analyzing the data were conducted using MAXQDA-2020. The analysis was performed following the Granheim and Lundman method in five steps [19]. In the initial step, after each interview, B.A. and N.N. transcribed the interviews on the same day. In the second step, J.Y.L. and B.A. carefully and repeatedly read the content of the interviews to grasp the overall meaning of the content. In the third step, J.Y.L. and B.A. systematically segmented the interview transcripts into semantic units and applied preliminary codes. In the fourth step, similar initial codes were grouped into broader categories. In the fifth step, latent patterns within the data were analyzed, resulting in the emergence of categories and subcategories. Eventually, the entire data analysis process was shared in a joint session to integrate the opinions of all the authors.

### Trustworthiness

Guba and Lincoln’s criteria were applied to enhance the rigor and quality of the results [20]. To enhance the credibility of the research, the researchers ensured diversity in sampling by including participants with varying economic, social, and demographic characteristics. Additionally, key informants with different areas of expertise were involved in the study. The overall understanding of the participants’ responses was summarized periodically during the interview and at the termination of the interview. The summarized understanding was then relayed to the participants, and any clarifications and corrections were made if necessary. A table of codes, subcategories, and categories with quotes was given to five participants at the end of the study to verify that the researchers had accurately reported their responses and experiences. To ensure confirmability**,** the data analysis and results were sent to four expert qualitative researchers. Any necessary revisions were made based on their feedback. To enhance dependability, all authors were involved in the process of data coding and analysis during multiple sessions throughout the study. They shared their opinions, and after approval was obtained from all the authors, the categories and subcategories were finalized. To increase transferability, several direct quotes from the participants’ interviews and the findings of the study were provided to several IHD patients who were eligible but did not participate in the study. They were asked if the participants’ experiences in this study accurately reflected their actual barriers to maintaining a healthy lifestyle and whether the findings resonated with their own observations or experiences.

### Ethical considerations

This study was ethically approved by Lorestan University of Medical Sciences (IR.LUMS.REC.1403.414) and was conducted in accordance with the Declaration of Helsinki. The participants were informed about the purpose of the study, research ethics, methods of data collection, and recording process. They were also assured that their information would remain confidential, and that they had the right to withdraw from the study at any time. Participants were also informed that all audio recordings would be deleted following the publication of the research findings. Written informed consent to participate was obtained from all participants.

## Results

This study included 31 patients with IHD (Table 1), two cardiology residents, three cardiology ward nurses, and three cardiologists as key informants (Table 2). Following the data analysis, four categories, seventeen subcategories, and 413 primary codes were identified and extracted (Table 3).

**Table 1:** Demographic Characteristics of IHD Patients.

Variables	Category	Frequency	Percentage
Age	< 50 years	4	12.9%
	51–69 years	20	64.5%
	≥ 70 years	7	22.6%
Gender	Female	13	41.9%
	Male	18	58.1%
Marital Status	Married	22	71.0%
	Single	9	29.0%
Education Level	Illiterate	12	38.7%
	High school diploma or below	15	48.4%
	Bachelor’s and Master’s degrees	4	12.9%
	Doctorate or higher	0	0.0%
Employment Status	Employed	15	48.4%
	Unemployed	9	29.0%
	Retired	7	22.6%
Place of Residence	Urban	21	67.7%
	Rural	10	32.3%
Duration of Illness	6-12 months	6	19.4%
	>1 year and ≤5 years	14	45.2%
	> 5 years	11	35.5%

**Table 2:** Demographic Characteristics of Key Informants.

No	Sex	Age (years)	Occupation	Education Level	Marital Status	Place of Residence
1	Female	34	Cardiology ward nurse	Master’s degree	Single	Urban
2	Female	36	Cardiology ward nurse	Bachelor’s degree	Married	Urban
3	Female	44	Cardiology ward nurse	Master’s degree	Married	Urban
4	Male	30	Cardiology resident	Doctor of Medicine	Single	Urban
5	Male	32	Cardiology resident	Doctor of Medicine	Married	Urban
6	Male	45	Cardiologist	Specialist in Cardiology	Married	Urban
7	Male	51	Cardiologist	Specialist in Cardiology	Married	Urban
8	Male	54	Interventional cardiologist	Fellowship in Cardiology	Single	Urban

**Table 3:** Categories, Subcategories, and Codes emerged from Interviews with IHD Patients and Key Informants.

Categories	Subcategories	Codes
**Socio-cultural barriers**	Taboos surrounding women’s sports	Unpleasant feelings about exercising in public places for women, cultural and clothing restrictions for women exercising in public, negative attitudes of older women towards wearing sportswear, women being prohibited by their husband or father from engaging in physical activity outside the home
	Misconceptions about sports	Perceiving physical activity as limited to hiking, considering exercise as something only for the young, believing that exercise is only for men, viewing exercise as inherently dangerous, and holding the belief that walking is an activity for the poor
	Fatalism	Believing that lifespan is in the hands of God, believing that the time of death is predetermined from the time of birth, believing that following a healthy lifestyle has no effect on lifespan
	Unhealthy dietary patterns and beliefs	Preparing large quantities of food at gatherings, serving fatty dishes at social events, high consumption of full-fat dairy products, perceiving eating less at gatherings as disrespectful to the hosts, the rise of fast food as a symbol of luxury, the habitual use of animal fat with a belief in its health benefits, the belief that red meat is heart healthy, the reluctance to stop consuming red meat and kebab due to fear of being perceived as poor by others
	Patterns and beliefs regarding the use of addictive substances	Smoking as a symbol of high social status, opium use associated with nobility, lack of societal stigma against opium use, considering the offering of opium to guests at gatherings as a sign of respect, the high prevalence of opium use in social gatherings
**Economic Barriers**	Economic crisis	Psychological stress due to sudden price changes, stress from children’s poor financial situations and unemployment, deep economic concerns overshadowing health priorities
	The high cost of a healthy lifestyle	The high cost of preparing separate meals from other family members, the high expense of gym memberships, the high cost of purchasing food for a healthy diet, the significant expense of teamwork in managing and improving patients’ lifestyles
**Individual Barriers**	Lack of prioritization of personal health	Prioritizing children’s favorite foods over recommended diets, prioritizing children’s issues over personal health
	Unhealthy food preferences	Preference for fatty and salty foods, dislike of vegetables, preference for full-fat dairy, preference for soda
	Lack of access to facilities and conditions for a healthy lifestyle	Limited physical activity due to illnesses or musculoskeletal pain, living in remote areas with limited access to healthy foods, lack of proper sports facilities, absence of opportunities for group exercise
	Personality traits	Low industriousness, tendency toward hedonic eating, being overly sensitive and easily irritated, having a high-stress personality, rigidity to change
	Low health literacy	Failure to understand the importance of maintaining a healthy lifestyle and the consequences of not following a healthy lifestyle, misunderstanding of healthy lifestyle recommendations, limiting adherence to a healthy lifestyle only to periods when symptoms are present, the belief that there is no need to follow lifestyle recommendations after stent placement or open-heart surgery, fears that stopping opium use will lead to stroke or paralysis, viewing opium addiction as hereditary, alcohol seen as heart-protective, recreational smoking perceived as harmless, weight perceived as innate and unchangeable
	Time constraints	Lack of time for physical activity due to work commitments, being occupied with household tasks and caring for children leaving no time for exercise, the multiple responsibilities of women in married life resulting in no time for exercise, not having enough time to prepare healthy meals due to a busy work schedule
**Healthcare-Medical Barriers**	Communication challenges in healthcare	Healthcare providers using overly technical language with patients, patients not being honest with healthcare providers due to fear of losing insurance coverage or legal consequences, failure to clearly ask about alcohol consumption due to concern that it could be misinterpreted by patients, and unrealistic strictness combined with a lack of professional integrity
	Management and infrastructure challenges in healthcare services	Low physician visit fees and extremely brief consultations, lack of a proper referral system, insufficient oversight of doctors
	Gaps in patient education and healthcare delivery	Lack of precise and quality education by doctors and nurses, recommendations limited to medication advice, oral education omitted due to inexperience or heavy workload, doctors’ belief in nurses’ responsibility for providing lifestyle recommendations, lack of coordination between doctors and nurses in delivering recommendations
	The perceived insignificance of lifestyle recommendations by patients	Patients’ interest in medical interventions, preference for shorter consultations over receiving lifestyle recommendations, and devaluing non medical advice from medical staff

### Socio-cultural barriers

A major obstacle for patients in adopting a healthier lifestyle is socio-cultural barriers. These include taboos surrounding women’s sports, misconceptions about sports, fatalism, unhealthy dietary patterns and beliefs, and patterns and beliefs regarding the use of addictive substances such as alcohol, cigarettes, and opium. Decision-making is greatly influenced by sociocultural factors, and decisions involving lifestyle and health are no exception. These decisions are formed and influenced by social norms, beliefs, and values.

### Taboos surrounding women’s sports

Many women who engage in physical activities in public places feel distressed and uneasy to continue physical activities in public places, mainly because such activities are tagged with social and cultural taboos. For instance, dress codes and cultural norms often restrict women from participating in sports in public places. Older women, in particular, might also develop negative attitudes toward dressing up in sporting costumes, as they fear negative reviews or sarcasm against them from society. Husbands and fathers may discourage women from exercising outdoors by imposing traditional gender role limitations. These pressures have further reduced in their potential to enjoy physical activities and have also made them feel ashamed of doing such activities. Here is a selection of quotes:

*“The place where we live is very small, and people know each other. It doesn’t have a good reputation for a woman to go out and exercise, so most women’s activities are done at home, usually in the form of housework.”*

(55-year-old woman)

*“Older women feel like if they go outside for a run or wear sports clothes, it’s as if they’ve committed a crime. That’s why fewer older women feel comfortable exercising outdoors.”*

(34-year-old cardiology ward nurse)

### Misconceptions about sports

Physical activities are limited by misconceptions, which serve as major barriers to individuals exercising and keeping fit. Older adults feel that sports are for the young; thus, they also view themselves as being excluded from participation. The problem is further compounded by safety concerns, as some individuals view physical activity as inherently risky. Additionally, walking is often stigmatized, and there is a belief that it is for poor people. Another common myth is that sports and physical activity are exclusively for men, which has discouraged women from participating. These misunderstandings have a significant role in reducing overall participation in physical activity, contributing to sedentary lifestyles and poor health outcomes in IHD patients. The following is a selection of statements:

*“I’m old now. I’m afraid something might happen to me during exercise. The risks of exercising at my age outweigh the benefits.”*

(80-year-old man)

*“Nowadays, life is like this. You come down, get in your car, and go. The situation has gotten to the point where even a 17-year-old drives around. For someone like me, it’s embarrassing to walk on the street. The neighbors and my family will see me.”*

(66-year-old man)

### Fatalism

A fatalistic attitude is very much influential in hindering people’s behavior toward healthy lifestyles. Many individuals believe that life expectancy is left to the discretion of a supreme being. Some IHD patients have internalized the widespread belief that all of these efforts are pointless in engaging in a healthy lifestyle given that such behaviors have no effect on their predetermined fate. These fatalistic presumptions reduce the likelihood of people adopting healthier practices or habits, thereby fostering a culture that discourages effective health management and promotes unhealthy lifestyle choices. Below are a few highlighted quotes:

*“I believe that lifespan is in God’s hands; no one’s life can be shorter or longer than what is meant to be. From the moment a person is born, it’s already determined how long they will live.”*

(61-year-old woman)

*“Everyone will die in the end, whether they follow a healthy lifestyle or not; they will live as long as God has destined for them. I prefer not to eat tasteless food during the lifetime God has given me.”*

(70-year-old woman)

### Unhealthy dietary patterns and beliefs

Food habits are heavily influenced by cultural beliefs and social behaviors, which also act as major barriers to healthier eating habits. At social gatherings, a substantial quantity of food is typically prepared, much of which consists of high-fat or oily dishes. Most individuals consume many local, high-fat dairy products but very few fruits and vegetables. The overconsumption of red meat makes maintaining a healthy diet more difficult. In addition, consuming less food during social gatherings is often avoided because of the fear of being perceived as impolite to the host. Fast food is now perceived as luxury food; and thus, individuals find it challenging to notice its negative health effects. There is also a common misperception of dietary fats; and therefore, the use of animal fats is still common and is believed to be a healthy option. Some patients believe that consuming red meat is good for their heart health and some are reluctant to reduce their intake of red meat or kebabs for fear of being perceived as poor. These cultural and social influences create significant challenges in the adoption of healthier eating habits. Here are a few selected quotes:

*“We used to buy oil from the store, from those liquid oils. But since I’ve heard these are genetically modified, I no longer buy them, and we consume animal fat instead.”*

(56-year-old man)

*“Here, people care a lot about what others think of them, and this makes it difficult for them to do many things. For example, they worry that if someone doesn’t eat meat or kebab, others might think they are poor and can’t afford it. So, they continue eating these foods just to avoid judgment.”*

(51-year-old cardiologist)

### Patterns and beliefs regarding the use of addictive substances

Traditionalistic cultural beliefs and misunderstandings, expressed as patterns and attitudes about addictive substances, give rise to behaviors that normalize and perpetuate substance use in a wide variety of social situations, which hinders the adoption of a healthy lifestyle among patients with IHD. For example, smoking is often seen as a sign of status and prestige, whereas opium use is traditionally associated with nobility and high social standing. There is also little negative social stigma surrounding opium use; to the extent that, in some gatherings, offering opium to guests is regarded as a gesture of respect. Addictant use, especially in social gatherings, has also normalized their usage to a great level. Here is a selection of quotes:

*“I think opium use here is higher than the national average because it’s somehow ingrained in the local culture. For example, it might be present at gatherings, or they might even offer it to guests as a sign of respect.”*

(34-year-old cardiology ward nurse)

*“Opium is a cultural issue here. People believe it’s associated with the elite, and there’s no stigma attached to using it.”*

(54-year-old cardiologist)

### Economic barriers

A significant challenge for patients in achieving a healthier lifestyle is economic barriers. These include the economic crisis and the high cost of maintaining a healthy lifestyle. Economic hardships shape decision-making, with financial constraints heavily influencing choices related to health and well-being, including a healthy lifestyle.

### Economic crisis

The economic crisis often leads to psychological stress due to the uncontrollable price rise and inability to plan finances appropriately. Financial crises and the resulting challenges, particularly those related to children, exacerbate psychological stress. Pressing economic issues overshadow health concerns, thereby slowing down actions concerning well-being when survival and having minimum economic stability become the priority. Here are a few selected quotes:

*“How can you not be stressed in these circumstances? For example, we’re farming, and suddenly everything goes wrong. Pesticide or fertilizer prices, for instance, can suddenly multiply. There are events that are hard to believe, and these things affect your mind, mood, and everything else. Our country still hasn’t been able to stabilize anything; for example, we plan based on the assumption that water costs one toman, but the next day, it’s a thousand tomans per liter. Most of my problems are economic.”*

(52-year-old man)

*“I’m always stressed about my kids. They’re farmers, and they work really hard, but their financial situation isn’t good.”*

(55-year-old woman)

*“The financial concerns are so overwhelming, and people are caught up in the daily struggles of life with the economic situation being so bad, that no one thinks about this issue. For example, no one says, ‘I’ll eat well now so that I’ll be better off in 5 years.’ No one even knows what tomorrow will bring.”*

(30-year-old cardiology resident)

### The high cost of a healthy lifestyle

The financial repercussions of living a healthy lifestyle deter many people from engaging in healthier habits. Many opt to eat the same meals as their family members, even if these options are not healthy, owing to the high cost of preparing separate meals. Furthermore, high costs associated with gym membership, increased expenses related to access to nutritionally rich foods, and the high cost of coordinated care and lifestyle interventions for patients present serious financial obstacles. As a result, these financial barriers significantly limit the ability of IHD patients to adopt and sustain lifestyle modifications essential for managing their condition and preventing further complications. Here is a selection of statements:

*“I haven’t followed the diet. I even eat fried food because I can’t cook two different meals, one for the kids and one for myself. It would be too expensive. I ate whatever the rest of the family ate.”*

(55-year-old woman)

*“My son goes to the gym and pays 250,000 tomans a month. If I want to go too, that would be 500,000 tomans just for the gym. Life has other expenses; it’s not all about the gym.”*

(57-year-old man)

### Individual barriers

Individual barriers create significant difficulties in pursuing a healthier lifestyle. These include neglecting self-care, preferring unhealthy foods, lacking access to the resources and conditions for a healthy lifestyle, having certain personality traits, having low health literacy, and having time constraints. Collectively, these factors create a complex web of challenges that hinder patients from adopting and maintaining healthier behaviors.

### Neglecting self-care

The neglect of self-care is due to prioritizing others’ needs over one’s own health, such as focusing on family members’ needs rather than personal well-being. This perspective leads individuals to neglect positive habits and practices that would help them secure better health in the long term. Below are a few highlighted quotes:

*“I got this illness, but thank God my kids are healthy. It’s unfair for them to have to eat tasteless food in their youth because of me.”*

(55-year-old woman)

*“I can’t bring myself to take away the money I could use to buy my child the things that make him happy, just so I can have the expensive oil or fish.”*

(64-year-old man)

### Unhealthy food preferences

Unhealthy eating preferences are often due to a liking for greasy, salty foods and sugary drinks, as well as a lack of interest in consuming healthier options, including vegetables. These choices lead patients to become more attached to food and drinks that are harmful to their health, making the transition to healthier eating habits challenging. Here is a selection of statements:

*“I’d rather live 70 years eating whatever I want than live 100 years on bland food and just vegetables.”*

(57-year-old man)

*“Even though I was told to eat less salt, I still ate a lot of it. I don’t like low-salt food or food without animal fat.”*

(70-year-old woman)

### Lack of access to resources and conditions for a healthy lifestyle

Access to resources and conditions for a healthy lifestyle is often hindered by factors such as restricted physical activity due to ailments and musculoskeletal pain, living in remote areas without access to healthy food, and a lack of suitable sports facilities or opportunities for group exercise. The presence of such barriers constrains people from engaging in health-promoting behavior and makes living a healthier lifestyle difficult and less reachable. Some noteworthy quotes are as follows:

*“I can’t exercise. My knee has developed wear and tear, and even my normal walking is painful.”*

(80-year-old woman)

*“We live in the mountains, so I can’t follow all the recommendations. For example, they say to eat low-fat dairy, but the milk and yogurt we get from our livestock are full-fat.”*

(55-year-old woman)

### Personality traits

Personality traits such as low industriousness, tendency toward hedonic eating, emotional sensitivity, high-stress personality, and resistance to change are important barriers to a healthier lifestyle for IHD patients. These traits can have a large influence whether an individual is able to embrace health-promoting behaviors. Tendency toward hedonic eating and low industriousness can lead to poor diet and a lack of physical activity, and emotional sensitivity and a high-stress personality can hinder effective stress management, which is a crucial component of a healthy lifestyle for patients with IHD. Resistance to change also creates difficulty in making long-term, healthy lifestyle changes. Below are some insightful quotes:

*“Food is an important part of my life, and I’m not willing to take this pleasure away from myself.”*

(67-year-old woman)

*“I’m already at an age where I’m set in my ways. Even if I wanted to, I couldn’t change now. The way I’ve lived so far is how I’ll continue living.”*

(62-year-old man)

### Low health literacy

Low health literacy results in a lack of knowledge about the importance of having a healthy lifestyle and the costs associated with not adhering to it. Misconceptions about healthy lifestyle recommendations and compliance with such practices only during periods of illness when symptoms are present, assuming that dietary rules are not needed after undergoing invasive treatments, such as stent installation or open-heart surgery, constitute part of the problem. In addition, certain beliefs contribute to the issue. To illustrate, some individuals believe that quitting opium could lead to stroke or paralysis, or that opium addiction is hereditary, similar to some diseases. There is also the misconception that alcohol consumption is beneficial due to its fat-cleansing effects and that recreational cigarette use does not harm heart health. These myths serve to prevent IHD patients from adopting and maintaining health-promoting behaviors, making a better lifestyle harder to achieve. Included here are some noteworthy quotes:

*“Everyone said that at your age, if you quit opium suddenly, you’ll have a stroke or become paralyzed. I thought that if that’s the case, it’s better to keep using it.”*

(69-year-old man)

*“I’ve heard that it’s good for fat; it cleanses the fats. For example, when I eat fatty food like kebab, I drink alcohol with it.”*

(52-year-old man)

### Time constraints

Time constraints are a major barrier to a healthy lifestyle. Extended working hours tend to leave little space for physical exercise or to prepare healthy foods. Most women, with several roles to play in their personal and professional lives, find it hard to allocate time for regular exercise. Such time limitations render the pursuit of a healthy lifestyle difficult and relegate well-being to a lesser priority. Here is a selection of quotes:

*“The doctor told me to eat low-salt and low-fat foods. Back when I had time, I used to cook for myself, and it was better. But now, I’m so busy that I just focus on filling my stomach. I don’t think about whether it’s healthy or not.”*

(42-year-old man)

*“Women have countless responsibilities at home besides working, and many of them, even though they care about their health, still don’t have time to exercise and take care of themselves.”*

(44-year-old cardiology ward nurse)

### Healthcare-medical barriers

Individuals with IHD face healthcare-medical barriers that make it difficult to adopt a healthy lifestyle. These include communication challenges in healthcare, management and infrastructure challenges in healthcare services, gaps in patient education and healthcare delivery, and the perceived insignificance of lifestyle recommendations by patients. These obstacles bar IHD patients from the achievement of a healthy lifestyle, as they restrict access to quality care, cause miscommunication between health care providers and patients, and give emphasis to medical interventions over lifestyle modifications.

### Gaps in patient education and healthcare delivery

Gaps in patient education and healthcare delivery are major obstacles to effectively guiding IHD patients toward a healthy lifestyle. These gaps include inadequate and low-quality education provided by healthcare providers, which is often limited to medication instructions without broader health guidance, including lifestyle recommendations. There is also a tendency for physicians to delegate educational responsibilities to nurses, while many patients show greater obedience to doctors’ recommendations. Besides, there are inconsistencies between the lifestyle advice provided by doctors and that provided by nurses, which further confuses patients, hence weakening the trust of patients in lifestyle recommendations. The following is a selection of statements:

*“When my illness started, I was hospitalized. The doctor didn’t tell me anything about what I should do considering my condition. But a nurse explained things to me for about two minutes when I was being discharged. She just spoke quickly and left, without giving me anything in writing. You can’t remember everything someone says in such a short time.”*

(46-year-old woman)

*“I haven’t seen doctors in the hospital discuss lifestyle modifications with patients. Maybe they think it’s solely our responsibility to address them. If doctors also talked about it, it would be much better because people trust their words more and tend to listen to them better.”*

(36-year-old cardiology ward nurse)

### Communication challenges in healthcare

Communication challenges between patients and healthcare providers significantly hinder the adoption of a healthy lifestyle among individuals with IHD, ultimately affecting their health outcomes. These include the use of highly technical jargon by health professionals, which complicates the understanding of the advice given to patients. Cultural and legal barriers may equally cause a patient to withhold true information from their healthcare providers, thus negatively impacting the guidance and support they receive for adopting a healthy lifestyle. Moreover, questions on sensitive topics such as alcohol consumption may not be asked clearly by healthcare providers, because of the fear of being misinterpreted. Unrealistic strictness coupled with a lack of professional honesty further complicates communication and delays the building of trust, which is essential for guiding patients toward a healthier lifestyle. Below are a few highlighted quotes:

*“I notice in the medical community, maybe it’s just in our country, that they often exaggerate to make you comply. For example, they say, ‘Don’t eat any fat or salt at all, or something terrible will happen to you.’ Then you go to another doctor who says a little is fine, or you start eating it yourself and realize that nothing major happens. This approach makes it hard to trust doctors in the future. Even if the next doctor is telling the truth about something serious, I might not believe them.”*

(61-year-old man)

*“If a patient’s alcohol use is noted in their medical history during admission, then yes, we definitely talk to them about alcohol. Otherwise, no, because many patients don’t like it. They feel like we’re judging them based on their appearance and assuming they’re alcoholics.”*

(34-year-old cardiology ward nurse)

### Management and infrastructure challenges in healthcare services

Various challenges regarding the management and infrastructure of health services negatively impact quality care, including lifestyle recommendations. Low consultation fees at clinics and short doctor visits further reduce the time for doctors to explain preventive measures and provide lifestyle guidance. A lack of referral systems makes patients seek specialists, even for minor health issues, which could have been treated by a general physician. This overwhelms specialists with the high volume of cases, thereby compromising the quality of care they can provide and further limiting the resources available to offer comprehensive lifestyle recommendations, which in turn makes it difficult for patients to adopt healthier habits. In addition, the failure to supervise physicians properly also undermines the effectiveness of the provided care since it results in the absence of quality of care minimum standards, thereby leaving it to personal preference. Here is a selection of quotes:

*“No one here has time for this kind of talk (lifestyle recommendations). Everywhere in the world, for example, a doctor might see 4 or 5 patients and spend 20 minutes with each. How many patients do we see in a day? Sometimes I see 45 or 50. I even remember a day when it reached 70. All of this is because the consultation fee is so low that you have to see so many patients to make it worthwhile. So, you’re forced to spend less time, and the first thing to be cut out is these things.”*

(45-year-old cardiologist)

*“There’s hardly any oversight. No one checks to ensure that, by law, you’re spending at least 15 minutes with each patient or providing the necessary recommendations.”*

(45-year-old cardiologist)

### The perceived insignificance of lifestyle recommendations by patients

The perceived insignificance of lifestyle advice by patients is also one of the major impediments to proper health care with regards to providing lifestyle recommendations. Most patients prefer medical interventions over advice on lifestyle changes, and they request procedures rather than preventive measures. In addition, physicians’ nonpharmacological advice is mostly underestimated by patients. Patients also prefer shorter visits that exclude lifestyle counseling instead of having to wait longer in the office for a more comprehensive consultation. This attitude results in fewer lifestyle recommendations being implemented in their daily lives, at the expense of their long-term health status. Here are a few selected quotes:

*“Patients don’t value it if a doctor takes the time to explain what to eat, what to avoid, or what actions to take. They believe the doctor’s role is to perform a procedure or, as they put it, ‘put them under some kind of machine’.”*

(54-year-old cardiologist)

*“If you sit in your office and spend 15 minutes with each patient to thoroughly explain and guide them, you’ll have patients outside the door shouting in frustration. It seems that neither we as doctors nor the patients visiting us have been taught to dedicate proper time for this process.”*

(45-year-old cardiologist)

## Discussion

This study sought to explore the barriers that IHD patients face in adopting a healthier lifestyles through a qualitative approach. The key barriers identified through this study include socio-cultural barriers, economic barriers, individual barriers, and healthcare-medical barriers.

Socio-cultural factors represent key barriers that may hinder individuals from engaging in healthier behaviors related to diet, physical activity, and general health practices. These factors are firmly rooted in cultural values, social norms, family ties, and community settings that can support or undermine the process of adopting better health behaviors.

A considerable number of female IHD participants in our research reported experiencing barriers to exercise due to the prevailing taboos regarding the participation of women in sports. As stated in previous research, these barriers are dominated by an interactive interplay between family values, religious and cultural restrictions, gender stereotypes, and limited or biased media representation [21–23]. These factors act to bar women’s entry into physical activity and the incorporation of better, more active living styles. Given that lifestyle modification is inherently challenging and often requires support from both family and the broader community, encountering resistance from these very sources significantly exacerbates the difficulty, rendering behavior change even more daunting and, in many cases, unfeasible for many women.

Misconceptions about sports, such as the belief that participation is restricted to a specific gender, age group, or type of activity, can act as barriers to engagement and promote sedentary behavior. These findings are consistent with those reported in previous research [24,25]. One novel contribution of this study was that certain misconceptions about sports can be linked to individuals’ perceptions of other individuals’ opinions of them while being active. For example, some participants believed that walking in public is primarily associated with lower socioeconomic groups and is perceived negatively in terms of social reputation.

Fatalism can act as an important impediment to lifestyle modification by inducing a perception of the inevitability of ill health and reducing motivation toward preventive lifestyle practices. A qualitative investigation of underprivileged men in Quebec who had suffered cardiovascular incidents revealed that fatalistic thought was one of the main obstacles to engagement in healthier lifestyles, as participants often continued lifestyles that conflict with health promotion recommendations [26]. Although some research indicates that the effects of fatalism can be context-specific and are not always limiting in every circumstance [27], our study predominantly revealed fatalism to be a limiting factor, resulting in reluctance toward lifestyle change in IHD patients.

The beliefs that unhealthy eating is inevitable, along with misinformation about what a healthy diet contains, can perpetuate maladaptive eating patterns and make positive behavioral changes less likely [28]. In our research, we noted a cultural trend in Lorestan in which it is traditional to prepare excessive amounts of food and serve high-fat foods at social events. Furthermore, guests tend to view eating modestly at such events as impolite, which reaffirms a social expectation that promotes overeating and establishes a cyclical obstacle to the acceptance of healthier eating. Another unhealthy dietary pattern that has become evident in recent years as a result of Iran’s ongoing economic challenges is the evolving attitude toward fast food. Once a cheap and nutritionally poor option, fast food has undergone an increase in price and is now perceived by some as a symbol of wealth. Therefore, even those who can afford better substitutes may choose fast food since the conventional view of it as only a cheap or budget-friendly one is no longer accurate. As noted earlier, concern regarding other people’s perceptions of one is a significant factor for a person’s intention to make lifestyle adjustments. In our study, avoidance of the consumption of foods such as kebab and red meat was in some cases avoided because of the social implication that such food avoidance is a sign of financial hardship.

Social and cultural norms strongly determine patterns and beliefs about the use of addictive substances, which can legitimate or stigmatize their use [29]. In Lorestan, especially for opium use, there is a sociocultural acceptance that promotes its normalization and increases the tendency for the use of addictive substances, to the extent that it is associated with nobility and even offered to guests as a sign of respect. However, the issue of addictive drug use far transcends lifestyle choice or the idea that one can simply control it with an act of will. Addiction involves sophisticated neurobiological adaptations, including gene expression changes and reward circuits, that compromise impulse control and generate severe cravings [30]. Stigmatization in this case is a double-edged sword. While intended to discourage the use of such substances, it can do harm by stigmatizing individuals with addiction, lowering their self-esteem, and discouraging them from seeking help [31,32].

To bridge these socio-cultural barriers, community-level interventions must focus on culturally tailored health education, promote media campaigns free of gender stereotypes, and involve local leaders in promoting healthy lifestyles. Assistance for women in the form of safe, easily accessible space for physical exercise and the creation of supportive peer and family settings can reinforce motivation for change. Emphasizing moderation over prohibition, pointing out low-cost, culturally appropriate healthy alternatives, and incorporating nutrition education as part of social events and community activities helps dispel misconceptions about diet. To battle fatalistic thoughts with successful stories and evidence-based treatment may foster a sense of agency and motivation for lifestyle change. Stigma reduction regarding addiction and respectful, nonjudgmental treatment are essential in terms of enabling recovery and encouraging help-seeking behavior.

Economic barriers play a critical role in hindering individuals’ ability to adopt healthier lifestyles, particularly among low-income populations. We observed in our study that the current economic crisis in Iran, along with its consequences such as inflation, high unemployment rates, and economic instability, has contributed to the induction of high mental stress in IHD patients. Such stress is particularly important, as it is one of the key factors that individuals with IHD must avoid in order to adopt and sustain a heart-healthy lifestyle. Another finding was that economic issues could become so severe that adopting a healthy lifestyle becomes a low priority and even a forgotten matter. In a study conducted in Greece, the level of depression tripled from 2008–2011, during which time austerity measures were initiated. Social isolation and depressive symptoms were also seen to significantly increased during this period, further demotivating individuals from engaging in healthy practices such as physical activity [33].

Financial constraints, as quoted by many IHD participants, greatly limit their ability to adopt a healthy lifestyle. Preparing separate meals specific for the dietary needs of IHD patients or purchasing healthier ingredients such as fish or olive oil is more expensive compared to routine food choices. Additionally, the cost of gym membership, which can help with more physical activity, is relatively high in comparison to family income, also restricting access to recommended lifestyle modifications. A teamwork approach in lifestyle interventions can increase motivation and provide beneficial emotional support, which can enable individuals to break through barriers and maintain healthier habits [34,35]. However, the financial demands of creating and implementing such programs make their widespread use in Iran difficult.

To overcome economic barriers to lifestyle change, interventions need to emphasize low-cost or community-supported solutions, including free public exercise programs, reasonably priced nutrition workshops, and subsidies for heart-healthy foods. Policy initiatives can aim at incorporating of lifestyle support within current healthcare services to balance extra costs. The promotion of home-based activities and peer-led support groups may also provide effective, low-cost alternatives to costly programs.

Individual barriers play a critical role in hindering lifestyle changes in IHD patients. Our results indicate that among the barriers in this domain is failure to prioritize one’s own health, particularly when individuals assign greater priority to child care responsibilities rather than their own health. This is in line with previous research, given that an intervention study involving 1,802 participants revealed that the explicit prioritization of some health behaviors significantly strengthened following these behaviors compared with not prioritizing them [36]. These results highlight that individuals are often involved in a number of conflicting demands at the same time, and without prioritization, health-enhancing action can fall behind more demanding or pressing concerns.

Some IHD patients in this study reported that not feeling any enjoyment while consuming healthy dietary choices such as vegetables or low-fat dairy, coupled with a desire for the taste of high-fat salty foods, full-fat dairy, and sweetened drinks such as soda, was an impediment to diet change toward a healthier one. Unhealthy food selection is often driven by taste appeal, convenience, social influences, and persuasive food advertising, all of which contribute to the overconsumption of calories and unhealthy eating behaviors [37]. One recurring problem identified in the literature is the general dislike of the taste of healthier food, which is a main hindrance to dietary improvement. A study by de Frel et al. reported that nearly 25% of survey respondents chose taste as a primary reason for not practising healthier food habits [38]. Even if individuals are aware of the importance of a healthy diet, regular fast food and sweets intake is still common. These findings suggest that deep-rooted tastes as well as food habits play important roles in health sensitivity in food choices [39].

Another individual-level barrier identified in our study was limited access to the necessary resources and conditions for maintaining a healthy lifestyle. This included the restricted availability of healthy food options and inadequate access to appropriate sports or physical activity facilities. These findings were consistent with prior research [40,41]. Another frequently cited barrier among IHD patients, particularly older individuals, was limited physical activity due to illness or musculoskeletal pain. IHD is common in older age groups, as observed in this study (12.9% under 50 years and 87.1% over 50 years). Musculoskeletal diseases such as knee osteoarthritis, lower back pain, and osteoporosis are more common in the same age group and can reduce willingness to perform physical activity due to associated pain, fatigue, and fear of symptom exacerbation [42,43]. Furthermore, patients with IHD can have restrictions on physical activity on the basis of complications of IHD itself or other accompanying health conditions [44]. These health problems can decrease physical capacity and render it more challenging to participate in regular exercise as part of an active lifestyle.

Many patients with IHD refer to certain personality traits they possess as obstacles preventing them from adopting a healthier lifestyle. Traits such as rigidity, low industriousness, and tendency toward hedonic eating were commonly mentioned. They have a negative effect through limiting the ability to modify established behaviors and to change for a better routine in terms of activity and diet. Additionally, in other studies, low conscientiousness, high neuroticism, low self-control, high impulsivity, low openness, low extraversion, and low agreeableness have all been noted as personality traits that can hinder the adoption and maintenance of healthy lifestyle changes [45–48]. Nevertheless, personality traits can be enhanced through interventions such as cognitive‒behavioral therapy and mindfulness, which may lead to better health behaviors and outcomes [46,49].

Many patients were unaware of the importance of maintaining healthy practices and believed that such measures were necessary only when symptoms such as chest pain or shortness of breath were present. After their symptoms improved, particularly following treatments such as coronary stent implantation or open-heart surgery, some felt that there was no longer a need to adhere to lifestyle recommendations. This lack of knowledge also extended to misconceptions about well-established harmful behaviors; for example, some patients believed that smoking or alcohol consumption had no negative effects or could even be beneficial. Additionally, a few participants attributed conditions such as overweight or opium use to hereditary factors, which may lead to the perception that making lifestyle changes is either ineffective or impossible. A large population-based study revealed that individuals with low health literacy are more likely to engage in several unhealthy lifestyle habits such as physical inactivity, poor diet, and smoking, than are individuals with higher health literacy. The association is stronger for those who are socially isolated, suggesting that social support may act as a buffer for some of the adverse effects of low health literacy [50]. Poor health literacy is also linked to lower understanding of health information, a reduced ability to follow medical instructions, and lower adherence to preventive health activities such as weight control, smoking cessation, and cancer screening, which can result in later stages of disease at the time of diagnosis and worse health outcomes [51,52]. Notably, the healthcare system has responsibilities extending beyond the provision of medical treatment or the performance of invasive interventions and equally encompasses the application of preventive interventions, which to a great extent depend on patient education and the filling of patients’ knowledge gaps regarding the disease and its prevention. This aspect is especially important in the context of IHD, in which adverse lifestyle factors contribute significantly to the development of the disease and related morbidity and mortality. Our study paid particular attention to this matter and will later be discussed as healthcare-medical barriers.

As mentioned by previous studies, time constraints can act as barriers to lifestyle change. In a primary care lifestyle intervention, lack of time was the most common perceived barrier to physical activity, and meal preparation time was one of the greatest difficulties for healthy eating [53]. Studies highlight that long working hours, nonstandard working schedules, and managing family duties allow minimal discretionary time for physical activity or the preparation of healthy meals. For example, working more than 40 hours weekly is highly correlated with the perception of time constraints hindering healthy eating, particularly in young adults [54]. Time constraints disproportionately impact young adults, individuals who live alone, and those with lower socioeconomic status [54,55].

Overcoming individual barriers requires a multifaceted approach involving tailored education, motivational interviewing, and behavioral counseling to enhance health literacy and promote healthy lifestyle change. Interventions such as taste adaptation techniques, resource facilitation, and exercise programs tailored for individuals with physical disabilities can also be effective. Additionally, psychological support through cognitive‒behavioral therapy and mindfulness training can help alter adverse personality characteristics and lead to the development of healthier habits in the long term.

One of the most important factors influencing lifestyle changes that has been less addressed in the literature is healthcare-medical barriers. Communication challenges in healthcare fall into this category. As seen in previous research [56], when healthcare providers counsel patients regarding their lifestyles using technical terms, especially in the context of low health literacy, it influences the understanding of the patients and renders efforts toward health education ineffective. In addition, a lack of transparency from both patients and medical staff, particularly in the context of alcohol consumption taboos, can hinder effective communication and prevent the delivery of appropriate lifestyle guidance. Medical staff mostly offer alcohol-related recommendations only to patients who explicitly disclose alcohol use to avoid potential misinterpretation. However, patients might resist reporting alcohol consumption for fear of legal consequences or social stigma, resulting in missing opportunities for effective counseling and intervention. A lack of transparency on the part of medical staff, such as in the recommendation of excessively restrictive or unrealistic lifestyle modifications, can discourage patients from adopting such measures. When patients subsequently receive alternative recommendations for lifestyle change and become aware that the previous advice was unrealistically restrictive or not evidence-based, this causes confusion and erodes patients’ trust in healthcare providers, therby reducing the effectiveness of subsequent lifestyle interventions.

Nearly all the key informants in our study mentioned management and infrastructure challenges as significant barriers to delivering proper lifestyle recommendations and interventions for IHD patients. All cardiologists stated that the low consultation fee demotivated them from devoting adequate time to lifestyle counseling for IHD patients because lifestyle counseling takes more time than simply prescribing medication. In addition, the lack of a proper referral system compels specialists to visit a vast number of patients, many of whom can be effectively treated by general practitioners. This burden of resources restricts the time spent per consultation, and specialists skip lifestyle counseling in their discussions. The intersection of these challenges with the lack of oversight over the quality of care by medical practitioners, especially the time spent per patient and the integration of lifestyle counseling alongside pharmacologic interventions, makes the challenge more daunting. Management and infrastructure barriers have also been noted by other studies. In one study, healthcare systems were faulted for emphasizing disease management and acute care more than preventive care and health promotion, limiting resource utilization and provision for healthcare for lifestyle intervention [57]. Moreover, organizational practices and policies were found to lack adequate incentives or facilitation for healthcare staff to participate in or refer patients to lifestyle change programs, lowering their effectiveness and intensity [58]. Traditional models of healthcare also tend to leave patients with minimal time for education during visits. Brief visits result in rushed explanations, minimal space to ask questions, and minimal time to address lifestyle changes. This inadequate ongoing support and education make it difficult for patients to maintain motivation and adapt their health plans, reducing the rate of successful lifestyle change [59,60]. The Iranian hospital settings showed a similar situation during the COVID-19 pandemic. Inefficiency in leadership and management and problems in service delivery hampered the provision of effective care and appropriate resource management for patients [61]. This thus demonstrates that barriers at structural and system levels pose a persistent challenge in the Iranian healthcare system, affecting both acute care and preventive services.

The majority of the patients complained that the lifestyle recommendations that were provided to them were incomplete and undetailed. In several cases, the advice was limited to a written handout with no verbal explanation and without ensuring that the patient understood or had the opportunity to ask questions. Some patients mentioned not receiving any recommendations at all, a point also confirmed by nurses, who attributed this to high workloads or the inexperience of some staff. It was also noted that this responsibility is frequently delegated entirely to nurses, despite the fact that patients often take doctors’ advice more seriously. The absence of a uniform structure for the dissemination of lifestyle guidance can lead to inconsistency in recommendations between providers and erosion of trust, and it may render recommendations not only unuseful but also harmful. Research has shown that programs incorporating interactive, personalized education, behavioral interventions, and peer support more effectively motivate patients to make changes. Group sessions, virtual support, and multidisciplinary teams empower patients over obstacles and promote a sense of community that is necessary to maintain motivation and positive change [62]. In contrast, generic or one-size-fits-all education is unable to address individual needs, attitudes, and challenges [63]. Providers may also face barriers while providing patient education regarding lifestyle, such as heavy workload, lack of training in health-promoting lifestyle advice, and the absence of the integration of health literacy strategies into routine care. Such system and provider-level restrictions further deepen the gaps in patient education and healthcare delivery [64]. Improving the quality of patient education also depends on the broader ethical and cultural development of healthcare providers. Evidence from Iranian paramedic staff suggests that education in bioethical principles, informed by religious teaching, can significantly enhance professional attitudes [65]. A recent study on Iranian nurses’ competencies concerning disaster risk management indicated that while the overall level of their competency is favorable, certain gaps still exist. This calls for continuous and structured training [66]. These findings further imply that culturally rooted and systematically reinforced education can improve the quality of care provided to patients.

The study also revealed that the perception of the insignificance of lifestyle advice among patients is an important barrier to lifestyle modification in patients with IHD. This perception is reflected in a preference for invasive procedures over preventive strategies, which favor shorter clinical visits rather than more comprehensive consultations and undervalue the importance of the lifestyle advice provided. This is primarily the outcome of a lack of initial education regarding the topic, which leads patients to either disregard the absence of lifestyle advice or, when such advice is given, perceive it as useless or irrelevant, thus decreasing the motivation of physicians who wish to emphasize these aspects and provide more comprehensive care. The literature suggests that the perceived insignificance of lifestyle recommendations is directly associated with lower adoption of preventive health behaviors [67]. A study done by Sánchez Urbano et al. indicated that patients are more likely to value and act on lifestyle advice when it is tailored, detailed, and delivered in a supportive context. In addition, the interpersonal quality of patients and caregivers stronlgy affects the perceived importance of lifestyle recommendations. Patients who feel that they are being listened to and understood are more inclined to value the importance of lifestyle change and attempt to act on it [68].

An integral solution to healthcare-medical barriers requires system and provider-level adjustments. Training health care providers in effective communication, including minimizing medical jargon and using health literacy strategies, can enhance patient understanding and engagement. Adding lifestyle counseling to routine care with established time, standardized processes, and interdisciplinary support may add consistency and credibility to advice. Incentivizing preventive care with remodeled consultation fees and improved referral mechanisms would motivate providers to invest more time in lifestyle counseling. Finally, the application of patient-centered education techniques, such as interactive counseling and individualized support, can improve patients’ motivation and adherence to lifestyle modifications.

Future research is required to establish the feasibility and efficacy of interventions on the barriers identified in this study. Community-based trials applying culturally tailored programs targeting women’s exercise barriers and fatalistic beliefs would be particularly useful. Research is also needed on how best to incorporate structured lifestyle counseling into routine cardiac care, including issues of cost-effectiveness and provider training to inform health system reforms. Longitudinal studies on the sustainability of lifestyle changes across socioeconomic status are needed. They can help determine which approaches yield long-term behavioral change. Quantitative research on the prevalence and relative impact of each category of barrier can also provide resource allocation and prioritization for prevention programs.

### Strengths and limitations

This study has multiple advantages that can increase the validity and reliability of the findings. Semi-structured in-depth interviews allowed in-depth contextual data collection, including complex barriers that patients with IHD face during lifestyle change. Triangulation of views was made possible by including both patients and key informants like cardiologists, residents, and nurses to have a broader understanding of the experience of these patients. Focusing on Lorestan Province, which has its own socio-cultural and economic conditions, helped to identify the local problems. To ensure methodological rigor, we used strategies like purposeful sampling for demographic variety, member checking, external expert review, and collaborative coding in line with Guba and Lincoln’s criteria. In addition, this study’s clear focus on and detailed capture of healthcare system-based barriers, an area that has received minimal attention in the majority of previous studies, is one of its most notable strengths.

Although the study has notable strengths, certain limitations must be considered. The qualitative and regional focus limits the generalizability of findings to other populations and settings. To address this, we provided rich context, detailed demographics, and direct quotes from participants to increase transferability. In addition, participants were purposefully selected, so there is potential for selection bias, particularly toward those who are more willing or able to share their experiences. We minimized this risk by ensuring variation in age, gender, socioeconomic status, and health literacy and including patients and healthcare providers from diverse backgrounds. Moreover, as data was self reported, the findings may be subject to social desirability or recall bias. In order to counteract this, we used neutral, nonjudgmental language during our private interviews and employed probing techniques to elicit candid, thorough answers. Family members’ and community leaders’ opinions, whose opinions could affect patient behaviors, were not included in the study. However, we partially compensated for this by including healthcare providers who observe patients’ family and community interactions.

## Conclusion

This study examined the barriers to IHD patients’ adoption of healthier lifestyles and categorized them into four broad categories, including socio-cultural barriers, economic barriers, individual barriers, and healthcare-medical barriers. Socio-cultural barriers in the form of taboos surrounding women’s sports, misconceptions about sports, fatalism, unhealthy dietary patterns and beliefs, and patterns and beliefs regarding the use of addictive substances were recognized as deeply entrenched issues. Economic barriers, including economic crisis and the high cost of a healthy lifestyle, also limit patients’ capacity to make progress toward a healthy lifestyle. Individual barriers, including low health literacy, unhealthy food preferences, time constraints, lack of prioritization of personal health, personality traits, and lack of access to facilities and conditions for a healthy lifestyle, also hinder lifestyle changes. Healthcare-medical barriers, such as communication challenges in healthcare, gaps in patient education and healthcare delivery, management and infrastructure challenges in healthcare services, and the perceived insignificance of lifestyle recommendations by patients, also undermined lifestyle interventions.

To minimize these barriers, there are a few options for possible interventions for implementation, such as community-level interventions for creating culturally relevant health education and low-cost sports facilities especially for women. Economic intervention programs that provide financial incentives for healthy foods and low-cost fitness programs may decrease economic barriers. Individual intervention programs such as tailored health literacy programs and health behavior counseling may diminish individual barriers. Health system reforms, such as adding structured lifestyle recommendations as part of routine care, enhancing training for health professionals, and improving referral pathways, may improve patient support. Although more research is needed to evaluate the feasibility and effectiveness of these measures, tackling these barriers in concert may enable IHD patients to make sustainable lifestyle changes and enhance long-term health outcomes.

## Supporting information

S1 File Interview Guide. (DOCX)

## References

[1] Zhang L, Tong Z, Han R, Guo R, Zang S, Zhang X, et al. Global, Regional, and National Burdens of Ischemic Heart Disease Attributable to Smoking From 1990 to 2019. J Am Heart Assoc. 2023;12(3):e028193. doi: 10.1161/JAHA.122.028193 36718860 PMC9973632

[2] Khan MA, Hashim MJ, Mustafa H, Baniyas MY, Al Suwaidi S, AlKatheeri R. Global epidemiology of ischemic heart disease: results from the global burden of disease study. Cureus. 2020;12(7):e9349. 10.7759/cureus.9349 PMC7384703 32742886

[3] Koolaji S, Sharifnejad Tehrani Y, Azadnajafabad S, Saeedi Moghaddam S, Shahin S, Ghamari A, et al. A 30-year trend of ischemic heart disease burden in a developing country; a systematic analysis of the global burden of disease study 2019 in Iran. Int J Cardiol. 2023;379:127–33. doi: 10.1016/j.ijcard.2023.03.012 36898585

[4] Sarrafzadegan N, Mohammmadifard N. Cardiovascular Disease in Iran in the Last 40 Years: Prevalence, Mortality, Morbidity, Challenges and Strategies for Cardiovascular Prevention. Arch Iran Med. 2019;22(4):204–10. 31126179

[5] Dai H, Much AA, Maor E, Asher E, Younis A, Xu Y, et al. Global, regional, and national burden of ischaemic heart disease and its attributable risk factors, 1990-2017: results from the Global Burden of Disease Study 2017. Eur Heart J Qual Care Clin Outcomes. 2022;8(1):50–60. doi: 10.1093/ehjqcco/qcaa076 33017008 PMC8728029

[6] Bisciglia A, Pasceri V, Irini D, Varveri A, Speciale G. Risk Factors for Ischemic Heart Disease. Rev Recent Clin Trials. 2019;14(2):86–94. doi: 10.2174/1574887114666190328125153 30919783

[7] Diab A, Dastmalchi LN, Gulati M, Michos ED. A Heart-Healthy Diet for Cardiovascular Disease Prevention: Where Are We Now?. Vasc Health Risk Manag. 2023;19:237–53. doi: 10.2147/VHRM.S379874 37113563 PMC10128075

[8] Rippe JM. Lifestyle Strategies for Risk Factor Reduction, Prevention, and Treatment of Cardiovascular Disease. Am J Lifestyle Med. 2018;13(2):204–12. doi: 10.1177/1559827618812395 30800027 PMC6378495

[9] Maino A, Sadeghian S, Mancini I, Abbasi SH, Poorhosseini H, Boroumand MA, et al. Opium as a risk factor for early-onset coronary artery disease: Results from the Milano-Iran (MIran) study. PLoS One. 2023;18(4):e0283707. doi: 10.1371/journal.pone.0283707 37074987 PMC10115251

[10] Taylor CN, Wang D, Larson MG, Lau ES, Benjamin EJ, D’Agostino RB Sr, et al. Family History of Modifiable Risk Factors and Association With Future Cardiovascular Disease. J Am Heart Assoc. 2023;12(6):e027881. doi: 10.1161/JAHA.122.027881 36892090 PMC10111537

[11] Gunnell AS, Einarsdóttir K, Galvão DA, Joyce S, Tomlin S, Graham V, et al. Lifestyle factors, medication use and risk for ischaemic heart disease hospitalisation: a longitudinal population-based study. PLoS One. 2013;8(10):e77833. doi: 10.1371/journal.pone.0077833 24147088 PMC3797723

[12] Ghodeshwar GK, Dube A, Khobragade D. Impact of Lifestyle Modifications on Cardiovascular Health: A Narrative Review. Cureus. 2023;15(7):e42616. doi: 10.7759/cureus.42616 37641769 PMC10460604

[13] Nielsen JB, Leppin A, Gyrd-Hansen DE, Jarbøl DE, Søndergaard J, Larsen PV. Barriers to lifestyle changes for prevention of cardiovascular disease - a survey among 40-60-year old Danes. BMC Cardiovasc Disord. 2017;17(1):245. doi: 10.1186/s12872-017-0677-0 28899356 PMC5596487

[14] Fifth Joint Task Force of the European Society of Cardiology and Other Societies on Cardiovascular Disease Prevention in Clinical Practice. European guidelines on cardiovascular disease prevention in clinical practice (version 2012). Europ J Prevent Cardiol. 2012;19(4):585–667. 10.1177/2047487312450228 22763626

[15] De Bacquer D, Astin F, Kotseva K, Pogosova N, De Smedt D, De Backer G, et al. Poor adherence to lifestyle recommendations in patients with coronary heart disease: results from the EUROASPIRE surveys. Eur J Prev Cardiol. 2022;29(2):383–95. doi: 10.1093/eurjpc/zwab115 34293121

[16] Ockene JK, Schneider KL, Lemon SC, Ockene IS. Can we improve adherence to preventive therapies for cardiovascular health? Circulation. 2011;124(11):1276–82. doi: 10.1161/CIRCULATIONAHA.110.968479 21911795

[17] Yang Y, Yuan Q, Wu C, Yang L. Barriers and facilitators to improved sedentary behaviour in coronary heart disease patients: a scoping review. BMJ Open. 2025;15(1):e088111. doi: 10.1136/bmjopen-2024-088111 39880456 PMC11781147

[18] Tenny S, Brannan JM, Brannan GD. Qualitative study. 2017. 29262162

[19] Graneheim UH, Lundman B. Qualitative content analysis in nursing research: concepts, procedures and measures to achieve trustworthiness. Nurse Educ Today. 2004;24(2):105–12. doi: 10.1016/j.nedt.2003.10.001 14769454

[20] Lincoln YS, Lynham SA, Guba EG. Paradigmatic controversies, contradictions, and emerging confluences, revisited. The Sage handbook of qualitative research. 2011. p. 97–128.

[21] He Y, Su G, Wang L, Qian H. Girls play basketball too? A study of the mechanisms of traditional social gender consciousness on female participation in contact leisure sports. Front Psychol. 2024;15. 10.3389/fpsyg.2024.1454003 PMC11496163 39444840

[22] Raza M, Ya Ling H, Hamdani SMZH, Hadier SG. Socio-Cultural Interest and Motivational Barriers for Female Sports Participation in Pakistan: A Comparative Study of Universities and Colleges. Sustainab Bus Soc Emerg Econ. 2022;4(2). doi: 10.26710/sbsee.v4i2.2393

[23] Isard RF, Melton EN, Macaulay CDT. Women’s sport and everyday resistance. Front Sports Act Living. 2023;5:1007033. doi: 10.3389/fspor.2023.1007033 37564915 PMC10410255

[24] Assembly NI. Barriers to sports and physical activity participation. Research Paper. 2010;18(10).

[25] Somerset S, Hoare DJ. Barriers to voluntary participation in sport for children: a systematic review. BMC Pediatr. 2018;18(1):47. doi: 10.1186/s12887-018-1014-1 29426310 PMC5810190

[26] Savage M, Dumas A, Stuart SA. Fatalism and short-termism as cultural barriers to cardiac rehabilitation among underprivileged men. Sociol Health Illn. 2013;35(8):1211–26. doi: 10.1111/1467-9566.12040 24266752

[27] Lennie TA, Frazier SK, Biddle M, Novak MJ, Casey B, Mudd G, et al. Abstract 19680: Fatalism does not decrease effectiveness of interventions to improve cardiovascular health. Circulation. 2012;126(suppl_21):A19680-A.

[28] Gherasim A, Arhire LI, Niță O, Popa AD, Graur M, Mihalache L. The relationship between lifestyle components and dietary patterns. Proc Nutr Soc. 2020;79(3):311–23. doi: 10.1017/S0029665120006898 32234085 PMC7663317

[29] Razaghi E, Farhoudian A, Pilevari A, Noroozi A, Hooshyari Z, Radfar R, et al. Identification of the socio-cultural barriers of drug addiction treatment in Iran. Heliyon. 2023;9(5):e15566. doi: 10.1016/j.heliyon.2023.e15566 37131427 PMC10149210

[30] Volkow ND, Michaelides M, Baler R. The Neuroscience of Drug Reward and Addiction. Physiol Rev. 2019;99(4):2115–40. doi: 10.1152/physrev.00014.2018 31507244 PMC6890985

[31] McLellan AT. Substance Misuse and Substance use Disorders: Why do they Matter in Healthcare? Trans Am Clin Climatol Assoc. 2017;128:112–30. 28790493 PMC5525418

[32] Hinshaw SP. Stigma Related to Substance Use and Addiction: The Long Journey Ahead-Commentary on Krendl and Perry (2023). Psychol Sci Public Interest. 2023;24(2):75–81. doi: 10.1177/15291006231202775 38095162

[33] Foscolou A, Tyrovolas S, Soulis G, Mariolis A, Piscopo S, Valacchi G, et al. The Impact of the Financial Crisis on Lifestyle Health Determinants Among Older Adults Living in the Mediterranean Region: The Multinational MEDIS Study (2005-2015). J Prev Med Public Health. 2017;50(1):1–9. 28173690 10.3961/jpmph.16.101 PMC5327683

[34] Taurio J, Järvinen J, Hautaniemi EJ, Eräranta A, Viitala J, Nordhausen K, et al. Team-based “Get-a-Grip” lifestyle management programme in the treatment of obesity. Prev Med Rep. 2020;19:101119. doi: 10.1016/j.pmedr.2020.101119 32461881 PMC7242875

[35] Wang Q, Chair SY, Wong EML, Qiu X. Actively incorporating lifestyle modifications into daily life: The key to adherence in a lifestyle intervention programme for metabolic syndrome. Front Public Health. 2022;10:929043. doi: 10.3389/fpubh.2022.929043 35979455 PMC9376606

[36] Conner M, Wilding S, Prestwich A, Hutter R, Hurling R, Harreveld F van, et al. Goal prioritization and behavior change: Evaluation of an intervention for multiple health behaviors. Health Psychol. 2022;41(5):356–65. doi: 10.1037/hea0001149 35467903

[37] Al Sabbah H, Ajab A, Ismail LC, Al Dhaheri A, Alblooshi S, Atari S. The association between food preferences, eating behavior, and body weight among female university students in the United Arab Emirates. Front Public Health. 2024;12. 10.3389/fpubh.2024.1395338 PMC11300260 39109159

[38] de Frel DL, Wicks H, Bakk Z, van Keulen N, Atsma DE, Janssen VR. Identifying barriers and facilitators to adopting healthier dietary choices in clinical care: a cross-sectional observational study. Front Nutr. 2023;10:1178134. doi: 10.3389/fnut.2023.1178134 38188877 PMC10767758

[39] Dong H-J, Brain K, Olsson M, Dragioti E, Gerdle B, Ghafouri B. Eating habits and the desire to eat healthier among patients with chronic pain: a registry-based study. Sci Rep. 2024;14(1):4705. doi: 10.1038/s41598-024-55449-z 38409442 PMC10897138

[40] Adams AK, Scott JR, Prince R, Williamson A. Using community advisory boards to reduce environmental barriers to health in American Indian communities, Wisconsin, 2007–2012. Prevent Chron Dis. 2014;11:E160. 10.5888/pcd11.140014 PMC4170726 25232747

[41] Fitzgerald N, Spaccarotella K. Barriers to a healthy lifestyle: from individuals to public policy—an ecological perspective. J Extens. 2009;47(1):2.

[42] Kalın AS, Aytur YK. Physical activity levels of individuals with chronic musculoskeletal disorders: Their relationship with barriers and facilitators. Musculoskeletal Care. 2023;21(3):797–805. doi: 10.1002/msc.1754 36876649

[43] Haas M, Boiché J, Chevance G, Latrille C, Brusseau M, Courbis A-L, et al. Motivation toward physical activity in patients with chronic musculoskeletal disorders: a meta-analysis of the efficacy of behavioural interventions. Sci Rep. 2024;14(1):18740. doi: 10.1038/s41598-024-67948-0 39138217 PMC11322353

[44] Joussain C, Joubert J, Laroche D, D’Antono B, Juneau M, Gremeaux V. Barriers to physical activity in coronary artery disease patients: Development and validation of a new scale. Ann Phys Rehabil Med. 2017;60(5):289–98. doi: 10.1016/j.rehab.2017.01.002 28216414

[45] Turiano NA, Pitzer L, Armour C, Karlamangla A, Ryff CD, Mroczek DK. Personality trait level and change as predictors of health outcomes: findings from a national study of Americans (MIDUS). J Gerontol B Psychol Sci Soc Sci. 2012;67(1):4–12. doi: 10.1093/geronb/gbr072 21765062 PMC3410684

[46] Wong MYC, Ou K-L, Chung PK. Healthy Lifestyle Behavior, Goal Setting, and Personality among Older Adults: A Synthesis of Literature Reviews and Interviews. Geriatrics (Basel). 2022;7(6):131. doi: 10.3390/geriatrics7060131 36547267 PMC9777641

[47] Burnos A, Skrobowski A. Temperamental and personality traits as factors related to changes in health behaviors and quality of life in patients with metabolic syndrome in Poland. Front Psychol. 2021;12. 10.3389/fpsyg.2021.709935 PMC8462662 34566787

[48] Tokarek J, Kapuścik A, Kućmierz J, Kowalczyk E, Karbownik MS. Personality traits and health-related behaviors in medical students facing a stressful event. Front Public Health. 2023;11:1256883. doi: 10.3389/fpubh.2023.1256883 37900039 PMC10602886

[49] Haehner P, Wright AJ, Bleidorn W. A systematic review of volitional personality change research. Commun Psychol. 2024;2(1):115. doi: 10.1038/s44271-024-00167-5 39616261 PMC11608366

[50] Wieczorek M, Meier C, Kliegel M, Maurer J. Relationship Between Health Literacy and Unhealthy Lifestyle Behaviours in Older Adults Living in Switzerland: Does Social Connectedness Matter? Int J Public Health. 2023;68:1606210. doi: 10.3389/ijph.2023.1606210 37876738 PMC10590881

[51] Stormacq C, Wosinski J, Boillat E, Van den Broucke S. Effects of health literacy interventions on health-related outcomes in socioeconomically disadvantaged adults living in the community: a systematic review. JBI Evid Synth. 2020;18(7):1389–469. doi: 10.11124/JBISRIR-D-18-00023 32813388

[52] Coughlin SS, Vernon M, Hatzigeorgiou C, George V. Health Literacy, Social Determinants of Health, and Disease Prevention and Control. J Environ Health Sci. 2020;6(1). PMC7889072 33604453

[53] Alageel S, Alhujaili M, Altwaijri Y, Bilal L, Alsukait R. Barriers and facilitators to adopting healthier lifestyle among low-income women in Saudi Arabia: A qualitative study. Health Expect. 2023;26(3):1202–12. doi: 10.1111/hex.13735 36806821 PMC10154786

[54] Escoto KH, Laska MN, Larson N, Neumark-Sztainer D, Hannan PJ. Work hours and perceived time barriers to healthful eating among young adults. Am J Health Behav. 2012;36(6):786–96. doi: 10.5993/AJHB.36.6.6 23026037 PMC3464955

[55] Pheasant H. Social, behavioural and other determinants of the choice of diet. Health Knowledge, United Kingdom. 2008.

[56] Murugesu L, Heijmans M, Rademakers J, Fransen MP. Challenges and solutions in communication with patients with low health literacy: Perspectives of healthcare providers. PLoS One. 2022;17(5):e0267782. doi: 10.1371/journal.pone.0267782 35507632 PMC9067671

[57] Subramaniam M, Devi F, AshaRani PV, Zhang Y, Wang P, Jeyagurunathan A, et al. Barriers and facilitators for adopting a healthy lifestyle in a multi-ethnic population: A qualitative study. PLoS One. 2022;17(11):e0277106. doi: 10.1371/journal.pone.0277106 36322596 PMC9629631

[58] Potthoff S, Kwasnicka D, Avery L, Finch T, Gardner B, Hankonen N, et al. Changing healthcare professionals’ non-reflective processes to improve the quality of care. Soc Sci Med. 2022;298:114840. doi: 10.1016/j.socscimed.2022.114840 35287065

[59] Pellisé F, Sell P, EuroSpine Patient Line Task Force. Patient information and education with modern media: the Spine Society of Europe Patient Line. Eur Spine J. 2009;18(Suppl 3):395–401. doi: 10.1007/s00586-009-0973-1 19381695 PMC2899323

[60] Barry A, Shahbaz A. The challenges and opportunities clinical education in the context of psychological, educational and therapeutic dimensions in teaching hospital. BMC Med Educ. 2025;25(1):154. doi: 10.1186/s12909-025-06711-z 39885476 PMC11780835

[61] Jabbari A, Salahi S, Hadian M, Khakdel Z, Hosseini E, Sheikhbardsiri H. Exploring the challenges of Iranian government hospitals related to Covid-19 pandemic management: a qualitative content analysis research from the nurses perspective. BMC Nurs. 2022;21(1):226. doi: 10.1186/s12912-022-01008-8 35962433 PMC9372986

[62] Ramli AS, Selvarajah S, Daud MH, Haniff J, Abdul-Razak S, Tg-Abu-Bakar-Sidik TMI, et al. Effectiveness of the EMPOWER-PAR Intervention in Improving Clinical Outcomes of Type 2 Diabetes Mellitus in Primary Care: A Pragmatic Cluster Randomised Controlled Trial. BMC Fam Pract. 2016;17(1):157. doi: 10.1186/s12875-016-0557-1 27842495 PMC5109682

[63] Ma Y, Cen W, Duan M, Yang J, Wang Y, Gao L, et al. Patients knowledge attitudes and practices regarding superficial fungal infections suggest public health and patient education are warranted. Sci Rep. 2025;15(1):15112. doi: 10.1038/s41598-025-98919-8 40301445 PMC12041239

[64] Dennis S, Williams A, Taggart J, Newall A, Denney-Wilson E, Zwar N, et al. Which providers can bridge the health literacy gap in lifestyle risk factor modification education: a systematic review and narrative synthesis. BMC Fam Pract. 2012;13:44. doi: 10.1186/1471-2296-13-44 22639799 PMC3515410

[65] Mohammadi MMD, Sheikhasadi H, Mahani SA, Taheri A, Sheikhbardsiri H, Abdi K. The effect of bio ethical principles education on ethical attitude of prehospital paramedic personnel. J Educ Health Promot. 2021;10:289. doi: 10.4103/jehp.jehp_708_20 34667789 PMC8459844

[66] Soltani Goki F, Farahmandnia H, Sabzi A, Taskiran Eskici G, Farokhzadian J. Iranian nurses’ perceptions of core competencies required for disaster risk management. BMC Emerg Med. 2023;23(1):84. doi: 10.1186/s12873-023-00853-3 37542294 PMC10403940

[67] Zhang P, Zhang L, Chen W. Patients’ perception of lifestyle advice as a mechanism between health shocks and health behaviours: Evidence from a longitudinal study in China. J Glob Health. 2024;14:04059. doi: 10.7189/jogh.14.04059 38515430 PMC10958586

[68] Sánchez Urbano RE, Paredes A, Vargas Chambi FR, Guedes Ruela P, Olivares DEV, Souza Pereira BT, et al. Reception of Dietary and Other Health-Related Lifestyle Advice to Address Non-communicable Diseases in a Primary Care Context: A Mixed-Method Study in Central Argentina. Front Nutr. 2021;8. 10.3389/fnut.2021.622543 PMC7873357 33585541

