# Supplementary material for: Multilevel obstacles to heart-healthy living in the context of Ischemic heart disease: A qualitative study
Source: PLoS One. 2025 Dec 9;20(12):e0338298. doi: 10.1371/journal.pone.0338298 (PMC12688121; doi:10.1371/journal.pone.0338298)
Supplement: S1 File — (DOCX) [file pone.0338298.s001.docx]

**Interview Guide**

The interviewing process should start by defining the study goals and purpose, participation procedures, data collection and audio recording procedures, and the researchers' and participants' roles. Informed consent must be documented for all participants prior to their involvement and at the beginning of the interview. Table 1 indicates the interview questions. The semi-structured nature of the interviews allows the interviewer the ability to go beyond the questions and pursue a more in-depth understanding of the participants' experiences and perspectives.

Table 1 Semi-Structured Interview Guide for Patients with Ischemic Heart Disease and Key Informants

| **IHD Patient Interview Questions** | 1. To what extent do you follow the healthcare team’s recommendations for managing your heart condition? |
| --- | --- |
|  | 1. What barriers or difficulties prevent you from fully adhering to these recommendations? |
|  | 1. How would you evaluate the quality and usefulness of the advice and support provided by your healthcare team? |
|  | 1. What are the primary challenges you encounter while trying to implement these recommendations in your daily life? |
| **Key Informants Interview Questions** | 1. What key topics do you typically discuss with IHD patients? What limitations or challenges do you experience in these discussions? |
|  | 1. Based on your experience, what are the main factors preventing IHD patients from adopting and maintaining a healthy lifestyle? |
|  | 1. In your view, what are the critical challenges patients face when attempting to manage their condition effectively? |
|  | 1. What are your thoughts on why some patients lack a proper understanding of what a healthy lifestyle means in relation to their condition and its importance? |
|  | 1. How would you describe the role of the healthcare team in supporting patients with IHD to improve their lifestyle and treatment adherence? |
